# ADTIDO: Detecting the Tired Deck Officer with Fusion Feature Methods

**DOI:** 10.3390/s22176506

**Published:** 2022-08-29

**Authors:** Chenghao Li, Yuhui Fu, Ruihong Ouyang, Yu Liu, Xinwen Hou

**Affiliations:** 1 College of Navigation, Dalian Maritime University, Dalian 116026, China; 2 School of Computer Science and Technology, Harbin Engineering University, Harbin 150009, China; 3 Institute of Automation, Chinese Academy of Sciences, Beijing 100045, China; 4 School of Artificial Intelligence, University of Chinese Academy of Sciences, Beijing 101408, China

**Keywords:** EEG, deck officer, fatigue detection, ECD-EEG fusion features, Bi-GRU neural network classifier

## Abstract

The incidence of maritime accidents can be significantly reduced by identifying the deck officer’s fatigue levels. The development of car driver fatigue detectors has employing electroencephalogram (EEG)-based technologies in recent years and made it possible to swiftly and accurately determine the level of a driver’s fatigue. However, individual variability and the sensitivity of EEG signals reduce the detection precision. Recently, another type of video-based technology for detecting driver fatigue by recording changes in the drivers’ eye characteristics has also been explored. In order to improve the classification performance of EEG-based approaches, this paper introduces the ADTIDO (Automatic Detect the TIred Deck Officers) algorithm, an EEG-based classification method of deck officers’ fatigue level, which combines a video-based approach to record the officer’s eye closure time for each time window. This paper uses a Discrete Wavelet Transformer (DWT) and decomposes the EEG signals into six sub-signals, from which we extract various EEG-based features, e.g., MAV, SD, and RMS. Unlike the traditional video-based method of calculating the Eyelid Closure Degree (ECD), this paper then obtains the ECD values from the EEG signals. The ECD-EEG fusion features are then created and used as the inputs for a classifier by combining the ECD and EEG feature sets. In addition, the present work develops the definition of “fatigue” at the individual level based on the real-time operational reaction time of the deck officer. To verify the efficacy of this research, the authors conducted their trials by using the EEG signals gathered from 21 subjects. It was found that Bidirectional Gated Recurrent Unit (Bi-GRU) networks outperform other classifiers, reaching a classification accuracy of 90.19 percent, 1.89 percent greater than that of only using EEG features as inputs. By combining the ADTIDO channel findings, the classification accuracy of deck officers’ fatigue levels finally reaches 95.74 percent.

## 1. Introduction

Human error is one of the leading causes of maritime fatalities (about 75–96%), with maritime fatigue being the major contributor [1]. It is recorded that maritime fatigue accounts for 23% of 98 maritime accidents [2]. During maritime navigation, deck officers are supposed to keep track of their surroundings. While in long-distance navigation, deck officers can hardly see land and other ships, making it easier to increase maritime fatigue. The International Maritime Organizations (IMO) are currently working hard to control the issue of seafarer fatigue. The 1995 amendment to the Seafarers’ Training, Certification, and Watchkeeping (STCW) Code included a claim for the officers’ cumulative effort and downtime. But deck officer fatigue is still a major issue. It is evident in the case of “Xingu 333” on 13 July 2021. In Guangzhou, China, a cargo ship collided with a bridge pier, resulting in a 9.9 million yuan immediate economic loss. According to the investigation, this accident involved only exclusive liability: the second officer of the steering ship failed to maintain a routine watch and failed to adjust the rudder in time to alter the ship’s course to line up with the navigable hole of the bridge, which directly caused the accident. The officer’s failure to perform steering maneuvers was directly related to their fatigue levels.

Based on maritime accident cases and the fact that maritime traffic accidents are often accompanied by significant casualties and property losses, this paper aims to develop an in-time method to pinpoint the worn-out deck officers. A practical tool that warns the captain to replace the tired deck officers is designed in the present work. The structure of this paper is listed as follows: Section 2 reviews related research. Section 3 introduces the details of experimental settings and the preprocessing approach for EEG signals. Section 4 provides information regarding ADTIDO. Section 5 discusses the experimental findings, such as feature extraction from fusion data and the precision of fatigue classification. Section 6 concludes the present work.

## 2. Related Works

Automatic fatigue detection is feasible by using physiological signals (e.g., EEG, electrocardiograph (ECG), and electrooculogram (EOG)). Due to its high reliability, EEG has been the main subject of research up to this point. The study on EEG-based fatigued driving in road traffic has discussed multiple EEG channel combinations, features (e.g., spectral entropy, wavelet entropy, delta rhythm, etc.) and classifiers (e.g., Random Forest, Decision Tree, K-Nearest Neighbor, etc.). Gao and colleagues [3] extracted wavelet entropy from driver EEG as features and developed a new complex network (RWECN). The driver’s EEG was divided into two categories: alert and fatigue. Compared with the traditional classification method based on wavelet entropy, it improves by about 5%. Han and colleagues [4] introduced complex network theory to study the evolution of brain dynamics under different EEG rhythms during simulated driving. It was found that the complex network characteristics of some channels and delta rhythm in the right brain changed with the deepening of fatigue. Hu and colleagues’ [5] work is applied to the fatigue identification of vehicle drivers, and a series of entropies such as SE, fuzzy entropy (FE), approximate entropy (AE), and spectral entropy (PE) are extracted as features. The RF classifier achieved better results (97.5%) in their work. Relevant studies have clearly defined classification standards regarding “alertness” and “fatigue”. The accuracy of EEG signals reduces with the number of classification standards increases because of the high noise level of EEG signals. Unlike vehicle drivers, deck officers have more working patterns, so their fatigue levels vary in different operations. The importance of multi-classification in determining the level of fatigue among deck officers cannot be overstated. According to [6], deck officers’ fatigue is significantly influenced by shift pattern, age, expertise in operating maritime craft, and the perceived difficulty of the voyage. Traditional fatigue research, however, is based on maritime accident news reports or accident evolution mechanism analysis in the maritime field. The limitation of this method is that it cannot detect human errors that did not contribute to the accident. Because collecting EEG signals is challenging, there are not many studies in the maritime field. Fan and colleagues [7] studied the role of the prefrontal cortex and functional connectivity during maritime operations. Researchers in Norway also proposed an algorithm that uses normalized EEG energy information to monitor the development of mental fatigue in maritime operations. The system was tested in a vessel simulator [8]. The sample sizes of relevant studies are insufficient and their performances are unstable in cross-individual tasks.

Another viable way for detecting fatigue is machine vision based on security camera footage. In the United States, a technique has been created to employ the PERcentage of eyelid CLOSure (PERCLOS) characteristics to calculate the fatigue level every three minutes [2]. By observing changes in the eyes, the video-based technique may intuitively detect driver fatigue. One significant disadvantage of this approach is the requirement for additional monitoring equipment. To address this issue, Arefnezhad with colleagues [9] discovered that almost 70% of the chosen biomarkers are increasing as PERCLOS grows during the driving test. This method makes it possible to predict the trajectory of PERCLOS using EEG signals in the following multiple seconds. Additionally, a linear relationship between Eyelid Closure Degree (ECD) and occipital EEG was confirmed. It presents a way to compute the ECD using EEG sensors instead of video-based methods [10].

The ADTIDO method proposed in this paper not only considers video as one of the features of fatigue to improve the precision of EEG-based fatigue level classification, but it also eliminates the need for additional video equipment, which is thought to be an efficient way to identify officers’ fatigue.

## 3. Materials

**Figure 1:**
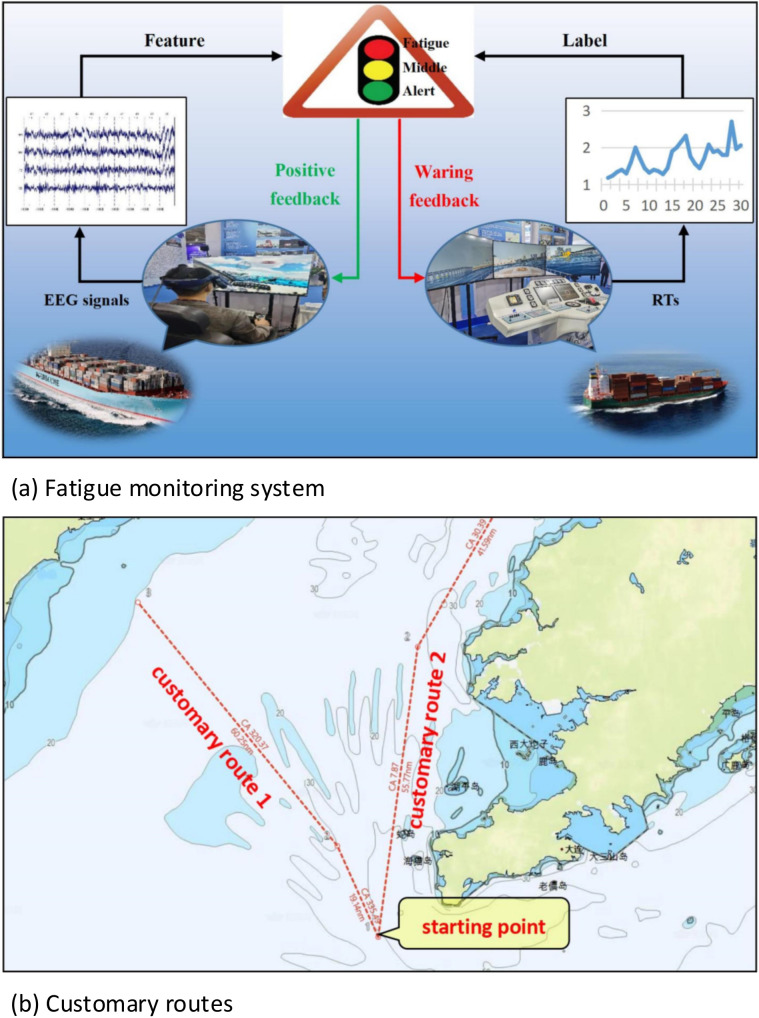
(**a**) The fatigue monitoring system. (**b**) Two different customary routes.

This section describes the method for gathering EEG signals and how to preprocess the data. It investigates two signal preprocessing methods to obtain different features and finally determines the fatigue level for an EEG signal according to reaction time (RT). The fatigue monitoring system (Figure 1a) in this paper will give deck officers additional feedback when reaching different fatigue levels.

### 3.1. EEG Signals Collecting

The subjects (deck officers) in a fatigue state are required to manipulate the ship, which may cause underlying safety problems. Therefore, the research collects the EEG signals of subjects performing navigation tasks on vessel simulators.

In the present work, 21 male students, ages 20 to 26, who were majoring in navigation technology and have a knowledge of manipulating ships, were invited to participate as our subjects (none of the participants had stayed up the night before). They were asked to perform various learning tasks for four consecutive hours to simulate a tired driving scenario. Workers who conduct the experiment thoroughly explain the experimental procedures to the participants (e.g., the operating methods of the ship simulator, how to wear the EEG collecting device correctly, etc.). After confirming that every subject is aware of the details of the experiment, subjects were asked to continue piloting the ship in a simulator for at least 90 min after lunch, and their EEG activities were well recorded during the navigation. Subjects were randomly assigned to two different routes (Figure 1b). Despite that both routes departed from the Laotieshan channel, the navigation environments were different. The first route, i.e., ocean navigation, was far from the coast, with few obstacles and less traffic flow. The second route, i.e., offshore navigation, was close to the coast and had more ships, islands, and other difficulties to navigate around.

The devices for EEG signal collecting—EPOC-Flex [11], which can completely cover the scalp with 32 channels, are provided by EMOTIV company. The EEG signals are recorded at a sampling rate of 500 Hz in the experiment. Meanwhile, the experimenter issues rudder orders to the subjects every three minutes (for example, “Port five” implies a rudder port of five degrees). As for reaction time (RT), experimenters track the interval that passes between the experimenter giving the subjects instructions and the subjects completing the activity.

### 3.2. EEG Signals Processing

**Figure 2:**
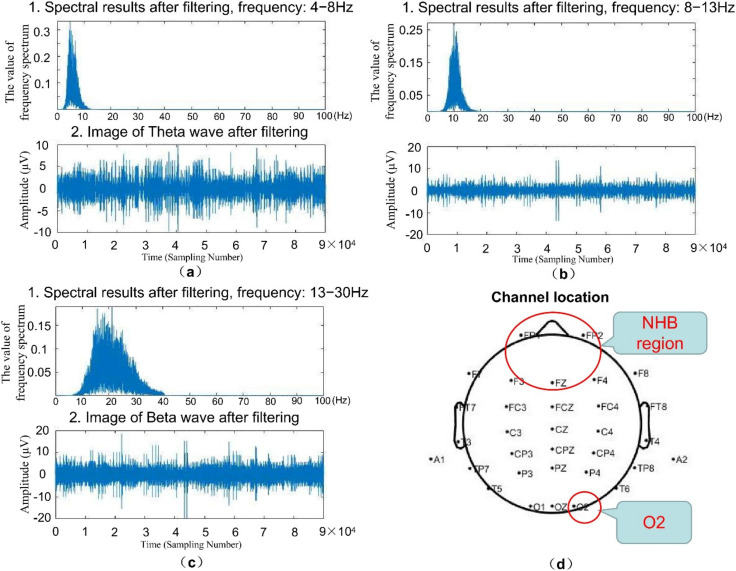
(**a**) Filtered spectral results and images of Theta waves in the O2 channel of subject 1. (**b**) Filtered spectral results and images of Alpha waves in the O2 channel of subject 1. (**c**) Filtered spectral results and images of Beta waves in the O2 channel of subject 1. (**d**) Subject 1’s channel location and the location of the selected channel.

The EEG signal processing consists of three steps. As illustrated in Figure 2, firstly, EEG signals were collected from four channels: three channels in the non-hair-bearing (NHB) region [12], and one channel on the right side of the occipital region [10]. The best fatigue classification can be achieved by EEG channels in the NHB region and the O2 channel which are located in the right side of the occipital region was found to be the best input feature for linear regression estimation of the ECD. The low-frequency drifts and 50 Hz noise were removed by using the band-pass filter at 1–40 Hz. In the second step, the highly associated components with the EOG were discarded by using Clean Rawdata and ASR (CRA). The three EEG stages of highest fatigue level, highest alertness, and moderate fatigue were reserved for the final step following baseline correction (elaborated in Section 3.3). It was noted that all the preprocessing methods mentioned above were executed using the EEGLAB toolbox [13]. Besides, the EEG signals collected from the O2 channel were filtered into separate frequency bands: theta (θ, 4–8 Hz), alpha (α, 8–13 Hz), and beta (β, 13–30 Hz) (Figure 2). This study topic is beyond the delta wave (δ, 0–4 Hz) and gamma wave (γ, 30–100 Hz) frequencies, which are mainly related to deep sleep and arousal effects, respectively [14].

Following the mentioned steps, the work segmented the EEG signals of the four channels into various samples with an interval time window of 1.8 s and obtain 100 samples for each channel, subject to different levels of fatigue.

### 3.3. Fatigue State Determination

In previous studies, researchers usually assigned the first and last windows of the EEG signals as the vigilant and fatigued signals, respectively [12]. In this paper, this kind of signal divination approach was regarded as unreasonable. On the one hand, it neglects the individual differences between subjects. On the other hand, the subjects may recover from weariness under continuous instructions [15], which means the most tired state of deck officers does not typically occur at the last windows of the signals.

**Figure 3:**
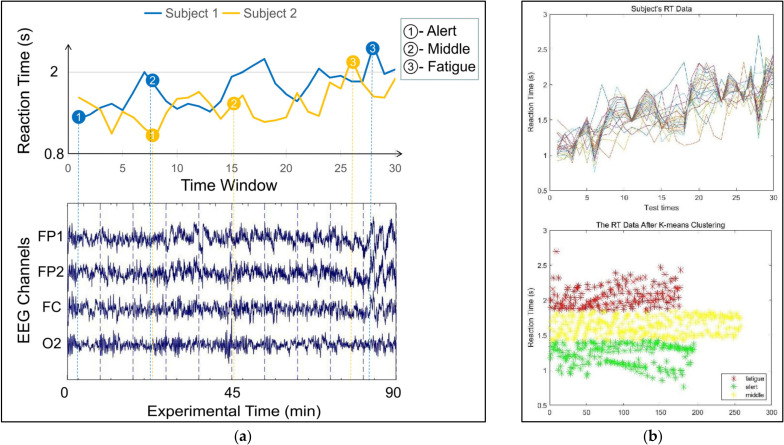
(**a**) The RT index was used to determine the navigating fatigue level. (**b**) Subject’s RT data after K-means clustering.

The IMO guidelines on fatigue define fatigue as follows: “a reduction in physical and mental capacity caused by physical, mental, or emotional exertion that may impair almost all physical abilities, including strength; speed; reaction time; coordination; decision making; or balance” [16]. In contrast to previous research, this paper distinguishes between the alert, middle, and fatigue EEG signals using the reaction time (RT). In this paper, the electronic stopwatch was used to record the reaction times of the subjects. All participants pressed the response button with their right hand before and after the steering order was given. Specifically, as illustrated in Figure 3a, this paper recorded each subject’s RT in fixed time windows and classified an EEG signal into one of three categories (alert, middle, or fatigue) based on the duration of RT. Subject 1 and Subject 2 drove on two routes, i.e., the ocean and offshore routes. Three-minute sections of EEG signals corresponding to each category were extracted as experimental data. The EEG signals with the shortest and longest RTs were recorded from the subjects in the alert and fatigue states, respectively, and those with the median RT were recorded from subjects who were in the midpoint of these two states. In this paper, the 30 RT data points of 21 subjects were subjected to K-means clustering to obtain three fatigue level categories, and 630 test points were clustered on the RT axis, as shown in Figure 3b. According to the experimental observation, the RT range of the fatigue state was 1.9 to 2.5 s, the RT range for the intermediate state was 1.4 to 1.9 s, and the range of RT during wakefulness was usually 0.8 to 1.4 s.

## 4. Methods

This section describes the details of ADTIDO, which consists of two steps: in the first step, effective features from EEG signals were extracted, including ECD feature and EEG features. In the second step, the category of an EEG signal was determined by feeding the data to a classifier.

### 4.1. Feature Extraction

This section introduces classifier features extraction consisting of two parts: ECD feature and EEG features extraction methods.

#### 4.1.1. ECD Feature Extraction

The PERCLOS is a video-based drowsiness detecting approach which monitors the alteration of subjects’ facial expressions, and indicates the proportion between the time that the eyelid covers beyond 80 per cent of the eyeball in a minute. Specifically, when the officer’s winking frequency increases quickly, PERCLOS judges that the officer is tired. The ECD value is the core of PERCLOS calculation, and PERCLOS is formulated as follows:

(1) $$PERCLOS=\frac{Time\left(ECD\geq 80\%\right)}{1\min}\times 100\%$$

Arefnezhad with colleagues [9] showed that the average Root Mean Square Error (RMSE) of PERCLOS estimated by biomarkers obtained from EEG was 0.117, and the average High Probability Density (HPD) percentage was 62.5%. Besides, 73% and 66% of the Theta and Delta powers were positively correlated with PERCLOS. Conventionally, the calculation of the ECD is based on image processing that monitors the facial postures of subjects. To distinguish the ECD extracted from the video-based method, this paper names the ECD extracted from the EEG signals “eECD”. Because the visual processing cortex is located in the brain’s occipital region, Li and Chung [10] claimed that there is a linear correlation between the EEG biomarkers of the O2 channel (Figure 2) and the ECD feature, in which the squared correlation coefficient R2 = 0.904. The R2 value is a measure of how the goodness of linear fit. If R2 = 1.00, indicates a perfect linear relationship. It motivates us to propose ADTIDO according to the linear relationship to extract the eECD feature. In terms of computational load, images require more storage space and longer processing time than signals. Apart from this, ECD extraction approaches have limited the developments in practical due to officers working at night or wearing glasses during the day. Therefore, eECD is considered convenient and efficient. We introduce how to obtain the eECD feature as follows.

The EEG data of the O2 channel is used to calculate EEG power percentages (θ, α and β power percentages) rather than absolute EEG power levels. By using a 1.8 s Hamming window, the EEG signal’s squared FFT magnitude is added to extract the EEG power. Then, the α power percentage (Per(α)) is calculated as the result of dividing the FFT power of the α EEG band by the sum of the FFT power of all three EEG bands (Equation (2), where $z_{i}$ = {θ, α, β}).

(2) $$\mathrm{Per}\left(\alpha \right)=\frac{Power\left(\alpha \right)}{\sum_{i=1}^{3}Power\left(z_{i}\right)}\times 100\%$$

A simple linear regression model is used to quantify the linear relationship between subjects’ ECD and EEG (Equation (3)) with slope $\hat{\beta }$ (=1.56) and intercept $\hat{\alpha }$ (=−31.37).

(3) $$\bar{ECD}=\hat{\alpha }+\hat{\beta }\times \mathrm{Per}\left(\alpha \right)$$

**Figure 4:**
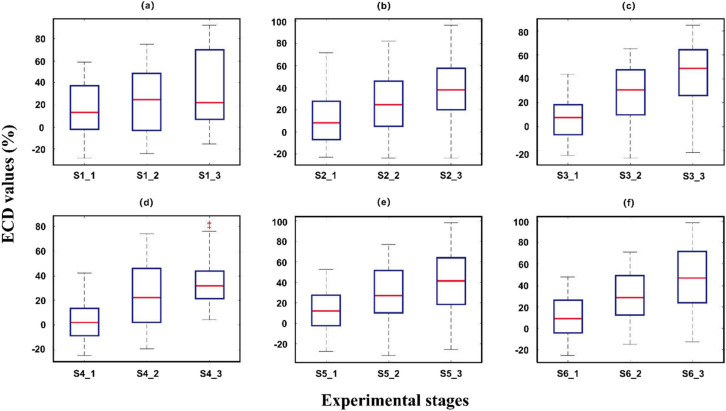
(**a**) ECD values of subject 1 in three periods of “alert”, “middle”, and “fatigue” respectively. The red line represents the median ECD value, and the upper and lower boundaries of the box are the upper and lower quartiles. (**b**) Subject 2’s ECD values. (**c**) Subject 3’s ECD values. (**d**) ECD values of subject 4 and the red cross stars indicate the outliers. (**e**) Subject 5’s ECD values. (**f**) Subject 6’s ECD values.

Because of the fitted model, some eECD values are negative. Overall, the eECD values are increasing in all three stages, which is consistent with the increase in fatigue associated with longer working hours. As the experiments in this paper were not set up with closed eyes, real driving situations with ECD reaching 80 are uncommon, so ECD values were chosen as features instead of PERCLOS. The eECD values of some subjects are shown in Figure 4. Finally, the present work normalizes the eECD values to both remove the dimension and intuitively reflect eyelid closure.

#### 4.1.2. EEG Features Extraction

The EEG signals can be regarded as non-stationary time series. The DWT makes up for the deficiency of Fourier decomposition in non-stationary time series to a large extent. The time-frequency localization and multi-scale refinement of the signal through scale transformation can better express the mutation and non-stationary part of the sequence. As a result, DWT is one of the ideal algorithms for processing and analyzing periodic signals. DWT of signal $s\left(t\right)$ is defined as:

(4) $$\mathrm{DWT}\left(m,n\right)=\int_{-\infty }^{+\infty }\frac{s\left(t\right)}{\sqrt{\left|2^{j}\right|}}\psi \left(\frac{t-2^{m}n}{2^{j}}\right)dt\;\left(m\in Z\right)$$

where ψ(·) is a wavelet basis function.

**Figure 5:**
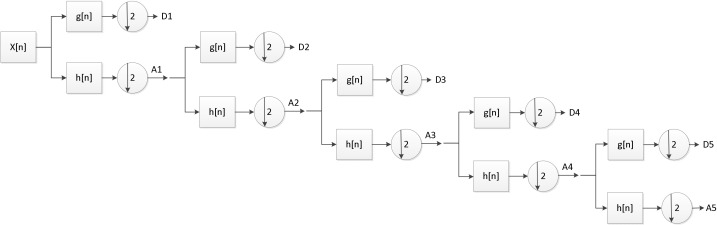
Fifth level wavelet decomposition of EEG.

The DWT’s heart uses a series of high-pass and low-pass filters to analyze signals at different frequencies. High-pass and low-pass filters are denoted by $g\left[\cdot \right]$ and $h\left[\cdot \right]$, respectively [17]. This paper decomposes the EEG signal into six sub-signals by a five-level decomposition of the db5 wavelet function, including D1, D2, D3, D4, D5, and A5. For the first level decomposition, results in D1 and A1 are the output of $s\left(t\right)$ bypassing $h\left(n\right)$ and $g\left(n\right)$, among that A1 is dominant in the frequency of the original signal. The approximation coefficient of each level is supposed to be decomposed continuously, and this process is repetitively executed four times to obtain the final sub-signals [18]. They are the frequency content of primary signals within the bands with fs/4–fs/2, fs/8–fs/4, fs/16–fs/8, fs/32–fs/16 and fs/64–fs/32, respectively, and the sixth subband A5 has a frequency range of 0–fs/64, among that the fs is 500 Hz. Each sub-signal in a different frequency domain on the officers’ current fatigue state, such as alpha waves (8 to 13 Hz) contained in the D5 (7.8 to 15.6 Hz) signals. In sleep research, changes in the alpha wave of EEG signal are considered the most reliable physiological marker of entering sleep [19]. The algorithm structure of five-level with wavelet decomposition for EEG signals is presented in Figure 5.

The EEG can be considered a zero-mean Gaussian random process. Therefore, it is appropriate to calculate the temporal characteristics of EEG using the Mean Absolute Value (MAV), Standard Deviation (SD), Root Mean Square (RMS) [20] and Shannon Entropy (SE) [21]. Then features of each sub-signal were standardized. A total of 19 EEG signal features were extracted, which are described in detail as follows:

The MAV: EEG shows strong randomness in amplitude, and the positive and negative amplitude is usually symmetrical. Absolute value operation converts the signal’s amplitude into a positive value, directly reflecting the degree of EEG change.

(5) $$\mathrm{MAV}=\frac{1}{N_{t}}\sum_{i=1}^{N_{t}}\left|x_{i}\right|$$

The RMS measures signal energy and is an appropriate method for calculating the mean EEG amplitude over a while.

(6) $$\mathrm{RMS}=\sqrt{\frac{1}{N_{t}}\sum_{i=1}^{N_{t}}x_{i}{}^{2}}$$

The SD: It is most commonly used in probability statistics to measure the degree of statistical distribution. It reflects the degree of dispersion between samples. The SD of a data set is the square root of the variance, where μ refers to the sample’s mean.

(7) $$\left\{\begin{array}{c} \sigma =\sqrt{\frac{1}{N_{t}-1}\sum_{i=1}^{N_{t}}{\left(x_{i}-\mu \right)}^{2}}\\ \mu =\frac{1}{N_{t}}\sum_{i=1}^{N_{t}}x_{i} \end{array}\right.$$

The SE: It has a major benefit in characterizing the randomness of signals and plays a vital role in non-stationary signal processing such as EEG. Complexity measurement is widely used in EEG studies of fatigue. Only SE of the D1 sub-signal is extracted because entropy change is evident in this band.

(8) $$H\left(X\right)=-\sum_{i=1}^{n}p\left(x_{i}\right)\log_{2}\left(p\left(x_{i}\right)\right)$$

This method has the benefit that the change of EEG signals can be represented in various frequency bands by multiple statistical features, and the increased sampling rate can lead to higher resolution. The extracted 19 features are strongly or weakly correlated with the current fatigue level of the deck officer, and the extracted 19 features are normalized to remove the dimensionality to obtain higher classification accuracy.

### 4.2. Feature Classification

**Figure 6:**
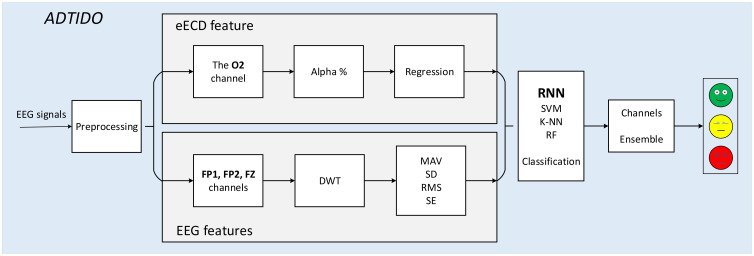
The flow chart.

In this experiment, various neural network classifiers were used based on recurrent neural networks (RNN) and machine learning classifiers such as SVM, K-NN, and RF for comparison. The flow chart is shown in Figure 6. In this paper, 7-fold cross-validation was adopted. The 21 sets of experiments were divided into training and testing sets in an 18:3 ratio, and results were taken an average of 7 times. By using this method, it can effectively reduce the effect of individual differences on the experimental results. The most effective Bi-GRU was selected as the classifier by evaluating the classification accuracy of each classifier based on the features gathered in this experiment.

#### 4.2.1. Classifiers Based on RNN

Long-Short Term Memory (LSTM) and Gated Recurrent Unit (GRU) are two special types of RNN. Compared with RNN, both LSTM and GRU can retain important features through various gates to avoid information loss during long-term propagation. The core of LSTM is the cell state. The EEG features are transmitted through cell states in a time-series pattern, and information is removed or added to cell states through “gates.” The GRU, a popular variant of LSTM, replaces “forget” gates and “input” gates with “update” gates. Compared with LSTM, GRU is more efficient with fewer parameters and can somewhat increase training efficiency.

**Figure 7:**
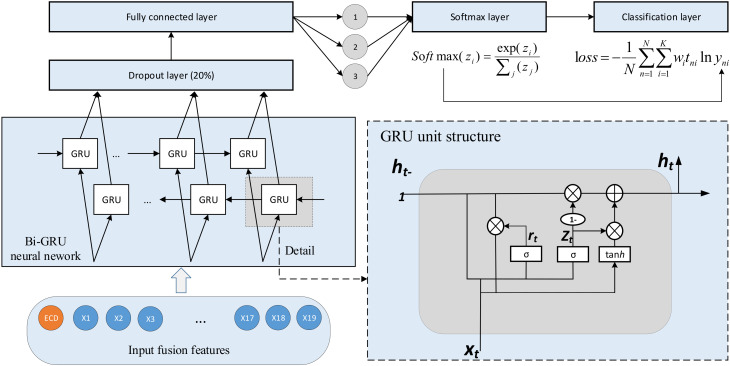
Bi-GRU neural network structure.

The GRU consists of an “update” gate ($Z_{t}$) and a “reset” gate ($r_{t}$), and $h_{t}$ is the memory unit. Its structure is shown in Figure 7. First, the state of $r_{t}$ and $Z_{t}$ can be obtained through the state ($h_{t-1}$) transmitted from the previous node and the input ($x_{t}$) of the current node:

(9) $$r_{t}=\sigma \left(W_{r}\cdot \left[h_{t-1},x_{t}\right]\right)$$

(10) $$Z_{t}=\sigma \left(W_{Z}\cdot \left[h_{t-1},x_{t}\right]\right)$$

After getting the gated signal, using the $r_{t}$ to get the reset data ($h_{t-1}*r_{t}$), then spline the reset data with the input ($x_{t}$), and scale the data to the range of [−1, 1] through an activation function (tanh), namely $h_{t}$:

(11) $$h_{t}=\tanh\left(W_{h}\times \left[h_{t-1}*r_{t},x_{t}\right]\right)$$

Through the $Z_{t}$ subsequently, which signal range is [0, 1]. The closer the gating signal is to 1, the more data is stored in memory. The GRU can use the same gated ($Z_{t}$) for both forgetting and selection.

(12) $$h_{t}=\left(1-Z_{t}\right)*h_{t-1}+Z_{t}*h_{t}$$

When faced with complex classification tasks and a large number of sample data, the bidirectional recurrent neural network can better extract sample features and improve the ability to fit complex samples. The Bi-GRU network [22] uses the upper GRU network’s output as the input of the following GRU network to train, which is suitable for the classification of EEG signals with large sample size. However, as the number of network layers deepens, the computation will be more extensive and time-consuming.

The forward and backward outputs of the Bi-GRU network at the time (t) are calculated by Equations (13) and (14). In the formula, $\vec{h_{t}}\in R^{h}$ is the output of the forward GRU network, and $\overset{\leftarrow }{h_{t}}\in R^{h}$ is the output of the rear GRU network.

(13) $$\vec{h_{t}}=\vec{\mathrm{GRU}}\left(x_{i},{\vec{h}}_{t-1}\right)$$

(14) $$\overset{\leftarrow }{h_{t}}=\overset{\leftarrow }{\mathrm{GRU}}\left(x_{i},{\overset{\leftarrow }{h}}_{t-1}\right)$$

“h” is the number of hidden layer units of the GRU network, and the hidden layer vector output ($h_{t}$) at the time (t) is determined by both forward and backward outputs. We show the $h_{t}$ in Formula (15), where $h_{t}\in R^{2h}$. Bi-GRU network consists of T moments, so the final implicit semantic encoding is shown in Formula (16).

(15) $$h_{t}=[\vec{h_{t}};\;\overset{\leftarrow }{h_{t}}]$$

(16) $$H=\left[h_{1},{\;h}_{2},{\;h}_{3},\;\ldots ,h_{T}\right]$$

In this experiment, the number of single-layer GRU or LSTM units is 128, the minimum training step size of the network is 600, the maximum training rounds is 30, and the learning rate is 0.005. This paper sets the drop rate of the dropout layer as 0.2, and the output of the fully connected layer is 3, which corresponds to the three fatigue levels. The Bi-GRU network structure is shown in Figure 7.

#### 4.2.2. Other Classification Algorithms

The SVM has the advantage of using the kernel function to map the 19 features to a higher dimension and search for the best separated hyperplane there, but it also has the drawback of making it easy to incorrectly classify the points near the hyperplane. The K-NN, another supervised learning technique, determines the K training samples in the training set that are closest to the supplied test samples based on the distance between them, and then makes predictions using the data from these K “neighbors”. This algorithm’s weakness is that it is sensitive to the balance of the samples’ distribution, and the training time complexity increases with increasing data volume. The classification result of RF is determined by voting on the classification results of all decision trees. The RF is good at processing high-dimensional data, and in comparison to SVM and K-NN algorithms, it is less susceptible to losing any data attributes. During the training of the decision tree, RF can detect the 19 feature dimensions that are retrieved. It can also rank the significance of the features. The drawback of this method is that parts of the actual findings are hidden because it is impossible to manage the behavior of numerous comparable decision trees inside the model [23].

## 5. Results and Discussion

### 5.1. Feature Extraction Results

The ECD features use a linear fitting model proposed by other scholars, and the differences in experimental individuals make the results subject to some error, which is likely to impair the classification accuracy of individual samples. For example, the sample 5400 shown in Table 1 has an eyelid closure of 26.5% in the fatigue state, and it is obvious that the fusion feature is not as effective as the single EEG feature at this time. In addition to eECD features, nineteen EEG features were expressed as A1, A2, A3, A4, B1, B2, B3, …, F1, F2, and F3; A4 represents the first subband’s SE, A1-A3 represents the first subband’s MAV, SD, and RMS, and A-F represents the six subbands decomposed by DWT.

In this paper, the fatigue levels of deck officers were defined according to the RT level, with the labels “1” for the awake state, “2” for the “middle” state, and “3” for the fatigue state. In addition, one of the purposes of the STCW Convention’s Watchkeeping Rules is to prevent crew fatigue. A principle in the rules states that “the officer in charge of the navigation shall be present at all times during working hours in the bridge control room or in a place directly connected therewith and shall be responsible for the safety of deck officers”. “The captain shall observe and judge the fatigue level of all deck officers”. When the captain finds that the senior crew member in charge of the watch shows signs of fatigue but can perform his duties, the captain shall arrange other personnel with sufficient energy to cooperate with him on the watch; “when the crew member has difficulty in ensuring a safe watch due to fatigue, he then should be replaced”. This paper contends that RT is a reliable and objective indication of whether a crew member who is showing signs of weariness can still execute navigating tasks.

The feature values of part of the training set are shown in Table 1, in which every three lines are the data of three channels of a sample.

### 5.2. Classification Results

**Figure 8:**
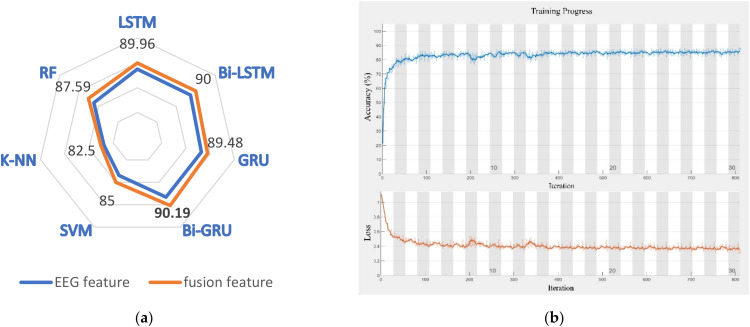
(**a**) The accuracy of classifiers under different features. (**b**) Training progress of BI-GRU network.

Overall, the fusion features outperformed the individual EEG features in terms of classification accuracy. The Bi-GRU had the highest accuracy of all of them at 90.19 percent, an increase of 1.89 percent over the single EEG feature and the training process of the Bi-GRU network is shown in Figure 8. When the classification accuracy of the ECD features in the Bi-GRU classifier was examined individually, it was only 56.33 percent. The key to the success of the ADTIDO method is to repeat the ECD values of each sample three times and assign them to the three EEG channels. The performances of other algorithms in fusion feature-based classification were: LSTM (89.96%), Bi-LSTM (90.0%), GRU (89.48%), SVM (85.0%), K-NN (82.5%), and RF (87.59%). The performance of the two features in each classifier is shown in Figure 8.

Compared to other classifiers, the RNN-based classifier was more effective, but the bidirectional network brings less improvement. The success of all the algorithms in this paper suggests that there is a strong correlation between the fatigue level of the deck officer and the features obtained using this method during the simulation. Additionally, this work also provides practical evidence for the potential utility of neurophysiological warnings for human error in ship operating environments.

This article replicates the algorithms of the other two works applied to this dataset for comparison. Gong and colleagues’ [17] work was used for the recognition of epileptic status. Both articles used DWT to extract MAV, SD, and RMS of different frequency bands as features, but the difference was that this paper selected eECD and SE as supplementary features, and used the Bi-GRU network, which was more suitable for deck officer fatigue classification, as a classifier. Compared with Hu and colleagues’ [5] work, this paper selected the most commonly used entropy (SE) as the fatigue feature. According to the 87.59% accuracy obtained by the RF classifier above, both the DWT algorithm and additional features can improve the classification accuracy. Based on the experimental data in this paper, the author used the same features and classifier to compare and found that the accuracy of the other two methods was lower than that of the method proposed in this paper. The resulting classification accuracy (CA) is shown in Table 2.

**Figure 9:**
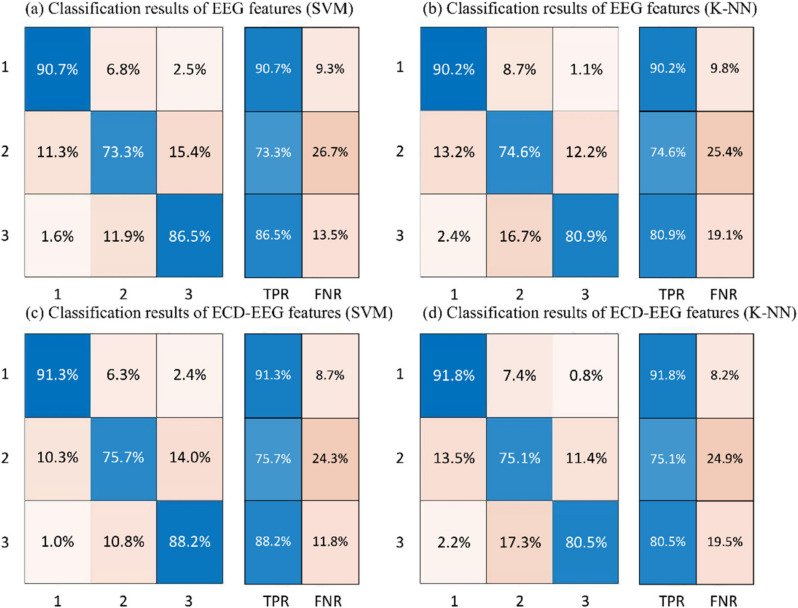
(**a**) Classification results of EEG features in the SVM classifier (Confusion matrix). (**b**) Classification results of EEG features in the K-NN classifier. (**c**) Classification results of ECD-EEG features in the SVM classifier. (**d**) Classification results of ECD-EEG features in the K-NN classifier.

The confusion matrix consisting of the classification results of SVM and K-NN is shown in Figure 9. The True Positive Ratio (TPR) describes the proportion of positive instances recognized by a classifier to all positive instances. When the single EEG feature was the input feature, the TPR of SVM was 90.7% for the “alert” state, 73.3% for the “middle” state, and 86.5% for the “fatigue” state. The TPRs of the three K-NN states were 90.2%, 74.8%, and 80.9%, respectively. When the ECD-EEG fusion features were used as input features, the SVM improved to 91.3%, 75.7%, and 88.2% for the three states, respectively. There was little change in the “middle” and “fatigue” states. It is easy to see that the “middle” state is more difficult to identify than the “alert” and “fatigue” states.

The prediction labels of the test set obtained by Bi-GRU are shown in Table 3. Each sample contains the prediction results of three channels, i.e., the fatigue status in every 1.8s time interval was determined by the three channels together. To make each sample correspond to only one fatigue level, the ADTIDO method was used in this experiment to correct the label prediction results. When ADTIDO considers that two or more of the three channel prediction results are the same, the classification result of that sample takes the majority channel prediction result (as shown in samples 1801 and 1804 in Table 3); when none of the three channel prediction results are the same, the classification is considered wrong (as shown in sample 3918 in Table 3). This experiment compared the corrected prediction results with the real labels and arrived at a final recognition accuracy of 95.74%.

## 6. Conclusions

Deck officers are more likely to experience driving fatigue because of their lengthy workdays, poor sleep quality, and complex sea conditions. The ADTIDO approach described in this paper intends to precisely identify the level of fatigue experienced by deck officers. It classified the extracted fatigue features with a 95.74 percent accuracy using Bi-GRU. Many EEG signal monitoring device designs today focus on comfort and miniaturization, which also allow more opportunities for real-world uses.

The ADTIDO method associates deck officer fatigue with RT values. When the deck officer is identified as “moderately fatigued”, the officer should adjust himself/herself by doing some relaxation exercises or chewing to stay focused. When the officer is identified as “fatigued”, the ship may be subject to “collision”, “touching”, “grounding”, etc. due to the officer’s failure to steer in time. In this case, the captain should arrange other personnel to cooperate with him on duty and use the RT index to judge whether the fatigued deck officer can perform the driving task [24]. By observing 21 groups of experiments, this paper concluded that when RT > 2.2 s (the average RT value of fatigue interval), the shift should be replaced in time. The experiment also finds that ocean trips will experience fatigue or weariness more quickly than nearshore excursions, allowing for more frequent shifts on ocean voyages. The experimental data currently available is based on simulation, so there is still a certain gap between the actual driving and the simulation. Additionally, because student volunteers in this experiment and deck officers differ from one another, it is more credible to conduct this test during a crew’s competency examination.

## Figures and Tables

**Table 1:** Partial feature extraction results.

	Channel	ECD	A1	A2	A3	A4	…	F3	Label
Sample _1	FP1	0.261	−0.834	−0.824	0.858	0.291	…	0.857	1
	FP2	0.261	−0.599	−0.586	0.634	0.071		0.643	1
	FZ	0.261	−0.882	−0.866	0.900	0.277		0.881	1
Sample _2101	FP1	0.467	−0.264	−0.227	0.281	0.003		0.274	2
	FP2	0.467	−0.299	−0.226	0.280	0.489		0.372	2
	FZ	0.467	−0.289	−0.252	0.307	0.040		0.359	2
Sample _5400	FP1	0.265	0.033	0.080	0.000	0.508		0.032	3
	FP2	0.265	0.601	0.666	0.553	0.109		0.555	3
	FZ	0.265	−0.840	−0.842	0.871	2.023		0.851	3

**Table 2:** Partial feature extraction results.

Author	Features	Methods	Results (CA)
This Article	eECD, MAV, SD, RMS, SE	DWT, Bi-GRU	**90.19%**
Gong, C. [17]	MAV, SD, RMS	DWT, P-NN	85.15%
Hu, J. [5]	SE, FE, AE, PE	RF	83.27%

**Table 3:** Classification results of Bi-LSTM and ADTIDO revision.

	Channel	Pred Label	ADTIDO Label	Real Label
Sample _1801	FP1	1	1	1
	FP2	1	1	1
	FZ	1	1	1
Sample _1804	FP1	1	1	1
	FP2	2	1	1
	FZ	1	1	1
Sample _3918	FP1	3	0	2
	FP2	1	0	2
	FZ	2	0	2
